# Cross-disciplinary insights on moral injury conceptualization and care: a qualitative study of expert perspectives

**DOI:** 10.3389/fpsyt.2026.1797654

**Published:** 2026-05-19

**Authors:** Falon T. Smith, Sarah M. Peterson, Maihan B. Vu, Melissa A. Smigelsky, Sophie A. Southerland, Aviva Shira Starr, Jason A. Nieuwsma

**Affiliations:** 1 Center for Health Promotion and Disease Prevention, The University of North Carolina, Chapel Hill, NC, United States; 2 Integrative Mental Health, Department of Veterans Affairs, Durham, NC, United States; 3 Department of Psychiatry, The University of North Carolina, Chapel Hill, NC, United States

**Keywords:** healthcare, intervention strategies, moral injury, psychological health, qualitative research, conceptualization, veterans

## Abstract

**Background:**

The term “moral injury” has been used to describe moral transgressions and their impact across populations. As a result of this broad application, interpretation of the construct and intervention strategies to address it have become increasingly nuanced and context-specific. This qualitative study explored how subject matter experts working in moral injury research and care across various populations view this complex phenomenon.

**Methods:**

Virtual focus group discussions were conducted with 33 researchers, mental health clinicians, and spiritual-care practitioners. Discussions explored the conceptualization of moral injury to inform implications for identification and care. Focus groups were digitally recorded, professionally transcribed, and thematically analyzed using qualitative software.

**Results:**

Experts highlighted the context-specific nature of moral injury, where interpretations and applications often vary according to their disciplinary backgrounds and the diversity of populations they serve. Based on their insights and experiences, key themes central to the conceptualization of moral injury and resulting implications for identification and care emerged. Conceptualization of moral injury focused on the development (i.e., progression from moral identity formation and disruption to a maladaptive response to moral pain and distress) and presentation (i.e., distinct features differentiating moral injury from other psychosocial-spiritual constructs). Implications for identification and care centered on the practical value and limitations of broad construct application, diagnostic considerations (e.g., the potential benefits and challenges of pathologizing moral injury), and moral injury-specific intervention and care recommendations, highlighting tailored approaches for addressing the multidimensional nature of moral injury.

**Discussion:**

Despite its conceptual complexity, moral injury is a phenomenon that resonates across diverse populations, contributing to its growing recognition and application across professional disciplines. Findings from this study reveal context-specific interpretations and applications of the construct, while also identifying core elements essential for understanding moral injury and informing effective intervention strategies. Overall, findings highlight the evolving, interdisciplinary nature of moral injury and suggest opportunities for tailored and integrative care strategies.

## 1 Introduction

Psychological constructs allow for a deeper understanding of abstract and complex human experiences and behaviors. By creating operational definitions of emerging constructs, we are better able to objectively assess the presence of a given construct and establish relevant intervention and treatment protocols. Constructs that evoke moral themes have long been used to describe experiences and outcomes in professional fields where individuals frequently encounter ethically challenging situations. In the early 1980s, moral distress was introduced to describe the psychological discomfort experienced by healthcare professionals facing ethical conflicts in the workplace (1). A decade later, Shay (2), a U.S. Department of Veteran’s Affairs (VA) psychiatrist, introduced the concept of moral injury. Through clinical observations of combat veterans, Shay documented symptoms that could not be adequately classified using Post-Traumatic Stress Disorder (PTSD) criteria. Drawing on parallels with Homeric literature, which depicts moral choices and ethical dilemmas inherent in war, Shay conceptualized moral injury as a distinct phenomenon resulting from the betrayal of “what’s right” by an authority figure or through one’s own actions in high-stakes situations. This framing foregrounded the moral and relational dimensions of war-related harm, distinguishing moral injury from fear-based trauma responses. Building on this foundation, Litz et al. (3) advanced moral injury as a formal psychological construct, highlighting gaps in clinical science and treatment for veterans experiencing feelings of shame and guilt resulting from exposure to potentially morally injurious events (PMIEs).

Preliminary conceptual and clinical care models established a foundational framework for moral injury research and intervention while acknowledging the construct’s complexity and the need for interdisciplinary approaches to fully define it. Consequently, efforts to operationalize moral injury have accelerated, particularly within military and veteran populations. The development of measures such as the Moral Injury Events Scale (MIES) (4), Moral Injury Outcome Scale (MIOS) (5), and Moral Injury and Distress Scale (MIDS) (6) has enabled the empirical investigation of PMIEs and associated outcomes. Concurrently, moral injury research has expanded beyond military contexts, to include healthcare (1, 7, 8), prison systems (9), first responders (10–12), refugees (13–16), journalists (17, 18), and educators (19, 20), among others; resulting in substantial heterogeneity in how the construct is defined, operationalized, and applied. Early clinical and trauma-focused models conceptualize moral injury as a betrayal of justice by a person in authority within a high-stakes environment, which disrupts moral identity, trust, and meaning-making, and leads to enduring guilt, shame, and relational disconnection (2, 3). Within this framework, social-functional perspectives conceptualize moral injury as a breakdown in moral and relational repair processes following moral conflict, particularly when opportunities for acknowledgement, accountability, or restoration are constrained. Alternatively, developmental & cognitive models emphasize the role of preexisting schemas, metacognitive beliefs, and the developmental context in shaping how individuals interpret and respond to PMIEs, as well as their exposure risk (19, 20). From this model, moral injury is a normal, adaptive response to moral transgression or betrayal that is shaped by one’s developmental experiences and cognitive and emotional schemas and metacognitions. The concern is when negative emotional schemas lead to suppression, impairments in functioning, and/or maladaptive coping. Other models emphasize the importance of the spiritual and betrayal-based component of moral injury, arguing for a holistic biopsychosocialspiritual approach that emphasizes moral injury as an existential and ontological wound with psychological, relational, cultural, and spiritual dimensions (21). Centrality of meaning, transcendence, and betrayal of values underpin this model, which calls for interdisciplinary care with spiritual and healthcare practitioners. Broader conceptualizations of moral injury, like structural models, frame moral injury from a systems perspective, one that is widely applicable and potentially experienced by everyone, while emphasizing the central role of collective (e.g., institutional, cultural, etc.) responsibility (22, 23).

While multiple, comprehensive frameworks have been employed to conceptualize moral injury, there is a divergence between models on what is affected, their developmental pathways, mechanisms of impairment, relative roles and importance of individual, relational, and systemic factors. Importantly, it is unclear whether the core assumptions of any single framework adequately capture how moral injury is conceptualized and addressed across disciplines and contexts. Identifying areas of convergence and divergence among subject matter experts (SMEs) across disciplines is critical for refining the construct, strengthening operational frameworks, and guiding effective models of care. The present qualitative study addresses this gap by exploring how SMEs across multiple disciplines conceptualize moral injury and apply the construct in practice. This qualitative study was conducted in partnership with VA investigators as part of a broader mixed-methods investigation of moral injury, its impact on post-9/11 veterans, and promising treatment approaches. Although the findings are intended to inform veteran care, the inclusion of diverse SMEs allows for the identification of shared conceptual foundations that may support existing theories and frameworks or identify needed modifications, contributing to ongoing efforts to develop a cohesive paradigmatic model of moral injury.

## 2 Materials and methods

### 2.1 Author positionality

The authors represent an interdisciplinary team including clinicians and moral injury researchers (two with Veterans Affairs affiliations), a clinical mental health provider, and public health and qualitative researchers at varying career stages. Focus groups were facilitated by team members with training in clinical psychology and marriage and family therapy, supported by a research assistant with a background in psychology and human development. We acknowledge that our professional roles and experiences may influence interpretation of the data. To mitigate this, we employed reflexive, team-based analytic approaches to ensure findings reflected the diversity of participant perspectives.

### 2.2 Study design

The present study was guided by a qualitative research design led by a collaborative team of researchers with expertise in qualitative methodology, public health, and mental health. Focus groups with SMEs from a variety of disciplines were conducted to explore experts’ perspectives on the foundational elements of moral injury that inform frameworks for effective intervention and care. The study was reviewed by the VA and determined to meet criteria for non-research operational activity. It was also reviewed by the University of North Carolina at Chapel Hill Institutional Review Board and deemed Not Human Subjects Research (IRB #24-2776).

A single-phase, qualitative descriptive design using focus group discussions was selected to capture SMEs’ direct accounts, facilitate multidisciplinary dialogue, and allow for exploration of conceptual and applied dimensions of moral injury. Based on social-ecological theory and relevant literature, the research team developed a semi-structured focus group discussion guide. The core topics of interest were shaped by research team members’ experience working within VA contexts and with moral injury care. Discussion guide questions were developed by the research team’s qualitative experts through a collaborative process. Initial drafts were informed by a thorough review of scientific literature and were iteratively refined based on feedback from the team’s mental health experts. Question formation drew from moral psychology, trauma studies, military sociology, and systems theory, and was intentionally designed to elicit conceptual, clinical, and system-level perspectives. Sample questions included: How do you primarily think about the concept of moral injury? What must be present across populations and circumstances to call something moral injury? What do people with moral injury need in terms of care and services? What do you see as the most important utility and value of the term “moral injury?”. These predetermined questions were used to guide discussions across focus groups; however, additional questions were asked where appropriate.

### 2.3 Participants

Purposive sampling was used to identify SMEs. An initial sampling frame of 42 experts was developed through independent review of peer-reviewed literature, citation networks, and established care programs frequently referenced in moral injury scholarship by team members with expertise in moral injury research, clinical care, and qualitative research. While the sampling frame included experts working with military, veteran, first responder, and healthcare populations, it was intentionally weighted toward individuals with direct experience working within U.S. veteran or service member context given the aim to inform veteran care. The sampling strategy sought to balance disciplinary diversity (e.g., researchers, clinicians, chaplains, and thought leaders) with contextual relevance. The original list of 42 SMEs was intentionally reduced to 35 to ensure that focus groups remained a manageable size while preserving the diversity of perspectives. The team carefully reviewed the list to include leading thought leaders across disciplines, while avoiding multiple representatives from a single organization or group.

Identified SMEs were contacted by telephone and email and asked to complete a digital survey to gauge interest, confirm eligibility, obtain consent, and identify availability for scheduled focus group sessions. Eligibility criteria included: (1) demonstrated expertise in moral injury (through research, care provision, program or policy development), (2) fluency in English, and (3) willingness and availability to participate in a 60-minute virtual focus group.

Thirty-three SMEs participated in the focus groups; one did not respond to multiple communication attempts, and one agreed to participate but was unable to attend the scheduled focus group session. Participants represented diverse professional backgrounds, and many held overlapping roles (see **Table 1**). The majority of participants self-identified as research experts (85%), though many also reported specialization in clinical (58%) and/or spiritual (46%) care. Seven participants (21%) reported having other professional expertise, including moral philosophy, healthcare ethics, education/training, interdisciplinary programming, and political theology. Just under one-fourth (24%) of participants reported holding veteran status. A little over half of the group (52%) indicated a current or prior affiliation with the VA (i.e., some SMEs were retired).

**Table 1:** Characteristics of focus group subject matter experts (N = 33).

	FG^a^1n=5	FG2 n=5	FG3 n=6	FG4 n=6	FG5 n=6	FG6 n=5	Total^b^ n=33
	n	n	n	n	n	n	n(%)
Veteran affiliation^c^							
Veteran	1	1	1	3	2	0	8 (24.2)
VA Affiliate	1	3	3	3	3	4	17 (51.5)
Professional role							
Research expert	4	5	4	6	4	5	28 (84.8)
Clinical care expert^d^	4	2	2	4	3	4	19 (57.6)
Spiritual care expert	2	1	3	4	3	2	15 (45.5)
Other^e^	2	1	3	1	0	0	7 (21.2)
Primary population of focus							
Active-duty service members/family	2	5	2	4	1	2	16 (51.6)
Veterans	5	4	5	3	5	5	27 (87.1)
First Responders/Police	1	2	1	0	0	1	5 (16.1)
Healthcare Providers	0	2	2	0	1	2	7 (22.6)
Clergy/Religious Leaders	1	0	3	1	0	0	5 (16.1)
Other^f^	0	0	1	0	1	2	4 (12.9)

^a^ FG, Focus Group.

^b^ Professional roles are reported for all participants (n=33); population of focus is based on available data (n=31). Participants could hold multiple roles or focus areas; percentages may exceed 100%.

^c^ VA, U.S. Department of Veteran’s Affairs.

^d^ Clinical care experts consisted of Psychologist (n=15) and Psychiatrist (n=4).

^e^ Other professional roles reported include moral philosopher, healthcare ethics, education & training, interdisciplinary programming, and political theology.

^f^ Other populations of focus reported include educators, legal professionals, and civilians.

While the final sample included a balanced distribution of SMEs across research, clinical care.

and spiritual care professions, most participants primarily focused on military (52%) and veteran (87%) populations. This was expected, given the origins of the moral injury construct, the substantial body of research on this population, and the study’s intent to inform VA care practices. A smaller number of SMEs focused on first responders, healthcare providers, or clergy (16%, 23%, 16%, respectively).

### 2.4 Data collection and analysis

Six, 60-minute virtual focus groups were conducted via Zoom software for live videoconferencing between March and April 2025. Each group was facilitated by two study staff members who were trained by the team’s qualitative experts to promote equitable participation among SMEs. Participants provided written informed consent electronically and additionally provided verbal consent prior to the recording of each focus group session. Discussions were audio- and video-recorded, professionally transcribed, and de-identified prior to analysis. Full focus groups, including post-discussion debriefing where facilitators reviewed notes and captured relevant nonverbal cues, lasted approximately 75 minutes. Reflexive memoing was used throughout analysis to identify and bracket assumptions and maintain analytic rigor.

Data analysis followed Bruan and Clarke’s multi-phase thematic approach, combining deductive and inductive strategies (24). First, senior qualitative researchers reviewed transcripts to ensure familiarity with the data. An initial codebook was developed deductively, informed by the focus group guide as parent codes (e.g., conceptualization, recommendations), with child codes generated inductively from results emerging from discussions with SMEs (e.g., frameworks, care strategies).

Two coders independently applied the codebook to an initial transcript using Dedoose (version 10.0.59) (25). The coders met to discuss and reconcile discrepancies, refine code definitions, and capture new or emergent codes. This iterative process was repeated across the remaining transcripts. Using Dedoose reports, code frequencies, and co-occurrence matrices, the PI examined relationships between concepts and grouped related codes into preliminary themes. Child codes were evaluated to determine whether they represented stand-alone themes or components of broader thematic patterns. Themes were refined through a systematic review of supporting excerpts, with attention to conceptual coherence, distinctiveness, and representation of the multidisciplinary perspectives present in the dataset.

Data saturation was assessed throughout the analytic process. The sample size of 33 SMEs split among six focus groups allowed us to assess data saturation across multi-disciplinary insights on moral injury conceptualization and care experiences. Consistent with grounded theory principles (26), saturation was defined as the point at which additional data collection no longer yielded novel or meaningful insights. For this study, our data saturation approach was based on our assessment of information redundancy in the intermediate and final stages of the data analysis process. Following initial coding and team-based discussion of the first half of the focus group transcripts, the qualitative research team noted convergence in emerging patterns along with areas of divergence. Analysis proceeded iteratively across remaining transcripts, with ongoing team discussions and comparisons within and across focus groups. Saturation was determined when no new themes emerged, and when additional data served to reinforce, rather than extend, existing thematic findings.

## 3 Results

Two overarching domains emerged from analysis: (1) conceptualization of moral injury; and (2) application of the construct in professional practice. Multiple themes and subthemes were identified, reflecting multidisciplinary perspectives.

### 3.1 Domain I: conceptualization of moral injury

SMEs conceptualized moral injury as a multi-layered, developmental phenomenon involving the formation of a foundational moral identity, exposure to moral transgression(s) that challenge moral identity, ensuing moral distress, and maladaptive responses that impair functioning. Distinguishing moral identity from other psychosocial-spiritual constructs was described as challenging but essential. Analysis revealed two main conceptualization themes and multiple subthemes.

#### 3.1.1 Theme 1: progressive development of moral injury

**Figure 1:**
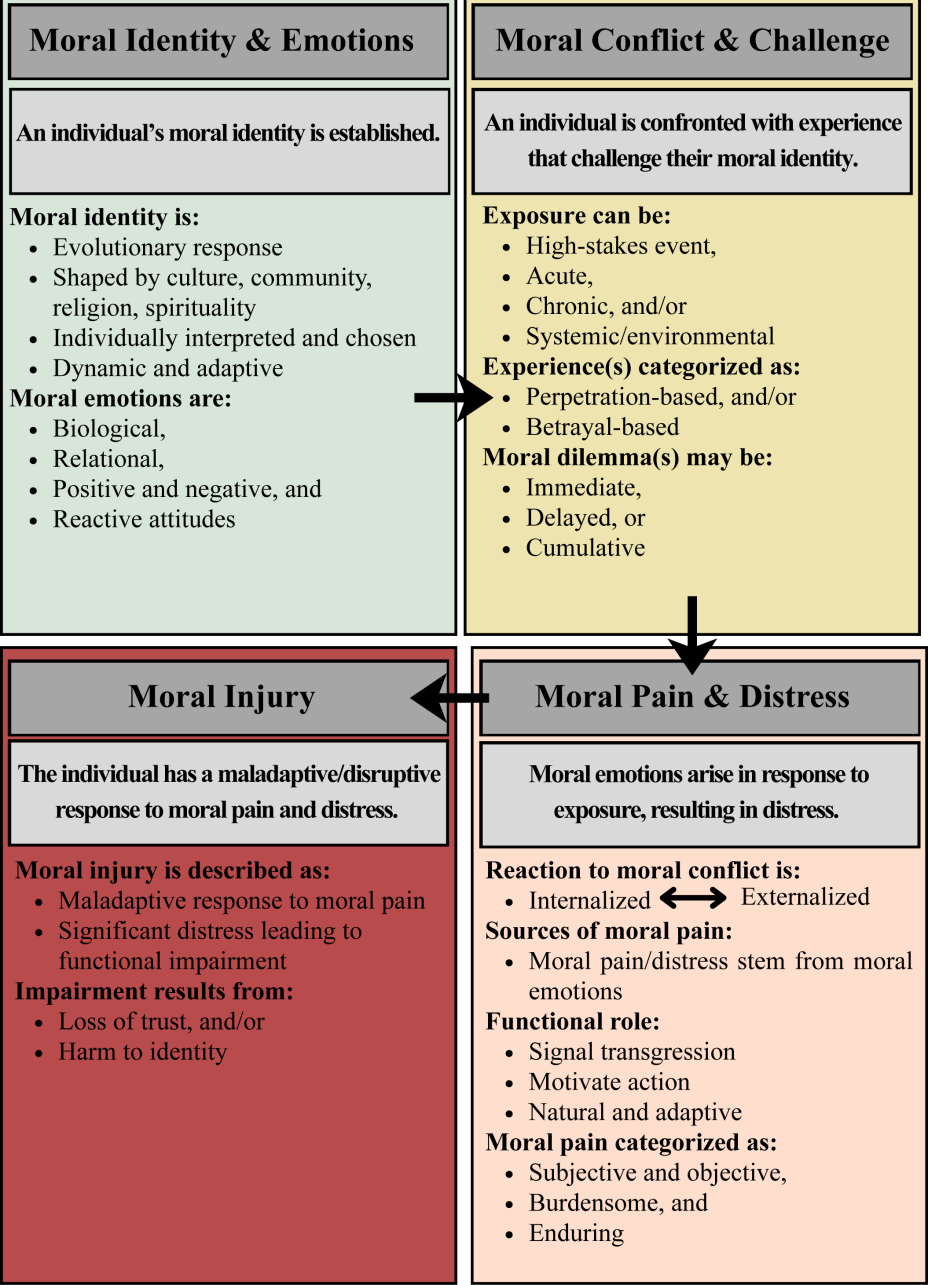
Subject matter expert proposed moral injury pathway.

SMEs consistently described moral injury as unfolding along a developmental pathway, beginning with an individual’s establishment of a moral framework, followed by exposure to a moral dilemma or harm that challenges one’s morals, and finally one’s reaction to the moral dilemma/harm, which could be considered healthy and adaptive or maladaptive, with the latter leading to moral injury (see **Figure 1**). Within this theme, four subthemes were identified.

##### 3.1.1.1 Moral identity is formed

SMEs discussed the development of morals as a foundational aspect to conceptualizing moral injury. They described moral identity as dynamic and shaped by multiple influences, including natural evolutionary processes, culture, and personal choice:

“I think that ties back into the culture, community, whether at large or particular communities—faith communities, different things that have a part in morally forming these individuals such that they’re then injured … they have a responsibility to help those people that have been morally injured partially due to how they’ve been morally formed as a part of the culture or a community.” (FG5, research and spiritual care expert 27).

They emphasized the role of moral emotions, which are biological, relational, and naturally occurring, in shaping moral identity and understanding moral injury:

“The moral emotions precede speech in the sense that emotions are signaled in our facial expressions, in our bodily posture, in our movements. We experience them internally. Moral emotions are relational emotions. I feel guilt when I’ve violated a relationship in some way,” (FG1, research and clinical care expert 3).

Taken together, these perspectives illustrate how differences in views on the development of moral identity and emotions shape understandings of moral injury.

##### 3.1.1.2 Moral identity is challenged

SMEs emphasized that moral injury arises when an individual is confronted with experiences that challenge their moral identity. There were varying opinions on what constitutes exposure that leads to moral injury. The necessity of high-stakes situations was discussed in four of the six focus groups. Some SMEs, notably those within the military context, endorsed a narrow view, defining moral injury as stemming from a single, high-stakes event, perceived as morally wrong and with potential for serious injury or death. However, the majority of SMEs challenged this constrained definition, viewing the high-stakes criteria for a PMIE as a limitation of its conceptual framework. One suggested that the high-stakes criterion may apply to military populations but is less relevant for humanitarian workers. Another noted that even within the military context, some veterans developed moral injury from events that did not meet the high-stakes threshold:

“(…)It seems like there’s been such a push to understand moral injury within a very high-stakes combat situation. Honestly, that is not what I’m seeing. I’m interviewing and working with veterans all the time. It’s research studies mainly and all of them had to deploy to Iraq, Afghanistan, but their moral injuries, there are a few of them came from a traditional combat situation.” (FG3, research expert 15).

Others emphasized chronic and systemic influences, which may unfold over time and contribute to moral injury beyond acute events:

“I also appreciated the nuancing of the concept of what is high-stakes because I think high-stakes can be a kind of drip, drip, drip, not a particular catastrophic incident or war or something like that. It can be a condition like poverty that finally wears you out or other kinds of what we would call morally distressing life contexts.” (FG3, spiritual care and interdisciplinary programming expert 13).

“I advocate for a kind of multi-focal lens where we are able to think about acute moral traumas, which would be sort of, again, specific around events, but in addition to that, think about chronic, environmental, intergenerational, and transgenerational dimensions.” (FG2, research and spiritual care expert 8).

Across focus groups, participants frequently described these experiences in terms of violation, whether of deeply held values, trust, or a sense of self. Building on this, some SMEs categorized experiences into two broad types: (1) perpetration-based, referring to when a person does something or fails to do something that violates their moral code; and (2) betrayal-based, referring to when a person feels their moral code has been violated by someone else (i.e., individual, organization, group), highlighting that these experiences are interpreted differently across context:

“(…) In my mind, perpetration-based moral injury is—obviously, a lotta overlap with these concepts of shame and guilt … When someone’s moral injury is really just strictly betrayal—which it’s not always so clean sometimes it’s both … Part of what’s complicated about our understanding of moral injury are these two subtypes. They seem, to me, a little bit of a byproduct of how the conversation has developed … Sometimes, in the conversations, I almost think the anger, the betrayal type moral injury drops out, at least in the setting I’m in. I wonder if, someday, as the definition refines itself, these will be two separate concepts.” (FG5, clinical care expert 26).

These insights suggest that moral injury can result from both acute and cumulative experiences, and its conceptual definition may need broadening to capture diverse populations and contexts.

##### 3.1.1.3 Moral pain and distress are experienced

SMEs explained that moral emotions are involved in the formation and communication of moral judgements, decisions, and beliefs. They are experienced internally in response to one’s experiences and events, particularly one’s own and others’ moral behavior. SMEs highlighted that moral pain and distress involve moral emotions that should not be pathologized, as they serve an appropriate, functional role signaling that something has been violated or broken, often in a relational context, which motivates actions to repair them. One expert explained:

“I like the language of adaptive response to moral pain … it’s about not avoiding moral pain, but working through it. In our work, moral pain is a lot about moral emotions and especially the negative moral emotions of guilt and shame and disgust and resentment. A big part of working through adaptively is to move away from what I would call maladaptive shame.” (FG4, research, clinical and spiritual care expert, veteran 17).

Building on these perspectives, SMEs spoke about moral pain as being both subjective and objective. They emphasized that what elicits moral pain is shaped by cultural and community contexts in which moral identity develops, making it burdensome and enduring. In one focus group, a couple of research experts emphasized the importance of individual perception in understanding moral pain, noting that what may seem transgressive to one person may not elicit pain in another. In response, another participant from the same group highlighted that some actions are objectively wrong. Multiple participants drew distinctions between internalized and externalized responses to moral pain and distress. Internalized responses are directed inward, where the person views themselves as having done something wrong. While this is often the case when a person does transgress (perpetration-based), they can also perceive themselves as having been responsible without having been. Moral emotions associated with this internalized belief may include, but are not limited to, guilt, shame, and self-condemnation. Externalized responses are directed outwards toward others, where the person perceives that other(s) have done wrong. This may be related to betrayal-based moral injury. Moral emotions may include, but are not limited to, anger, blame, and aggression.

Across focus groups, a recurring theme was that moral pain leading to moral injury often arises from irreversible actions that violate a person’s core beliefs, values, or sense of right and wrong; pain that does not simply fade with time but persists, resurfacing and shaping one’s identity and moral functioning in enduring ways.

##### 3.1.1.4 Moral injury is the maladaptive and disruptive response to moral pain and distress

While moral pain and distress were largely viewed as normal, adaptive responses to moral violations, moral injury was described as an impairment of functioning (i.e., moral, psychosocial, spiritual, or relational) that emerges when responses to moral pain become maladaptive. Participants emphasized that this is the point where moral emotions shift from being signals that prompt growth or repair to becoming overwhelming and all-consuming, preventing individuals from living in alignment with their core values:

“I see the moral pain as a signal that something’s been violated, something’s broken—oftentimes, relationally—and a motivator to take actions to improve that situation. I think what I classify as moral injury ends up being the behaviors I engage in to fix that moral pain don’t work. I choose badly.” (FG1, research and clinical care expert 3).

“What’s important to distinguish is, in my opinion, moral pain from moral injury is the way we respond to that pain. Are we able to listen to it, learn from it, maybe, and grow from it, heal from it, reconcile? Whatever the case may be the adaptive constructive response. Or do we suffer inside of it? Do we struggle with it? Do we buy into thoughts of self-condemnation, or do we become entangled with disconnecting ideas about other people in the world?” (FG4, research and clinical care expert 19).

For many SMEs, functional impairment was identified as a critical criterion for characterizing moral injury. One expert explained that while distress can also be adaptive, barriers to resolving distress can prevent recovery and healing. When those barriers become insurmountable, the result is “clinically significant distress and functional impairment” (FG2, research expert 9). Expanding on considerations of functional impairment, one SME noted:

“I think of the concept in two ways … In both ways, it has to do with an impairment of moral functioning. The first way is an impairment in moral functioning that arises from unfair distribution of appropriate moral pain, where somebody is stuck in isolating shame, for example, or holding more than their fair share of a moral burden that belongs to a community. The second way is an impairment in moral functioning that arises from the inability or unwillingness to experience appropriate moral pain. That occurs when people are stuck in moral disengagement, and they refuse, or they’re just incapable of experiencing appropriate moral emotions.” (FG4, research, clinical and spiritual care expert, veteran 17).

Another highlighted the relational dimensions of this impairment:

“Moral injury is really identified when someone is experiencing impairment in psychosocial functioning in their relationships, their spiritual practice, and their self-care in relation to how they respond to that distress. The distress isn’t the problem. It’s their behavior and response to it.” (FG5, research and clinical care expert 25).

Some SMEs emphasized that this impairment stems from violations of core identity or loss of trust, differentiating moral injury from moral pain by its lasting disruption to moral, relational, or psychosocial functioning. Across discussions, breakdowns in relational cohesion and erosion of trust were described as consistent features of this disruption, observed across disciplines, populations, and cultural contexts at both an individual and systemic level.

#### 3.1.2 Theme 2: challenges to identifying moral injury

Discussions around the presentation and assessment of moral injury, how it compares across populations and contexts, and how it is related to and distinct from existing mental health and spiritual constructs, were central to its conceptualization. Within this theme, two subthemes were identified.

##### 3.1.2.1 Overlap with other psychosocial-spiritual constructs

Some SMEs expressed difficulty distinguishing moral injury from other mental health issues given that they can occur simultaneously (e.g., moral injury and PTSD) and share similar psychological symptoms. While similarities between PTSD and moral injury were acknowledged, most SMEs shared the opinion that the two are distinct constructs. PTSD was characterized as a “fear-based” response to a clearly defined traumatic event, whereas SMEs described moral injury as a shame-based experience with an enduring sense of betrayal. While SMEs noted that moral injury can arise from a traumatic event, they emphasized that a traumatic event is not a defining criterion, as one expert described:

“Sure, there are these big-picture events. ‘I was driving the truck. I was at the guard post. I was in a hospital’ whatever these big, I guess for lack of a better term, more criterion A PTSD-ish events. In moral injury, they’re definitely there. There’s also the just slow burn and erosion of morale and culture that I don’t know that—well, the PTSD measures don’t capture” (FG6, research, clinical and spiritual care expert 31).

Multiple SMEs across focus groups highlighted that both PTSD and moral injury involve harm, but that the nature of that harm differs. It was suggested that identifying and defining the types of harm unique to each could serve as a method to differentiate between the two. Multiple SMEs noted the potential significance of moral injury being used to refine the current understanding of PTSD, with one describing their work with veterans where they found that “moral injury events were far stronger predictors of PTSD than fear-based events” (FG2, research and clinical care expert, veteran 6). They shared that others have reported similar findings, suggesting that moral injury may be a predictor of PTSD or an indicator of complex PTSD.

##### 3.1.2.2 Variability and subjectivity in symptom expression

SMEs described moral injury symptoms as including guilt, shame, unworthiness, helplessness, rage, or suicidality, and behaviors such as dissociation, isolation, hyperarousal, or violence. SMEs referred to moral injury as a “shame or guilt” syndrome, highlighting the central role of shame:

“Too many persons who struggle with an experience of moral injury—because my conversations suggest that shame is isolating. We know that. Whether I’m ashamed of what I did or I’m shamed because someone I trusted betrayed that trust, it’s isolating.” (FG1, research, spiritual care, education and training expert 4).

Several SMEs noted that moral injury exists on a spectrum: mild experiences of shame and guilt can be functional, whereas more severe experiences can lead to significant dysfunction:

“The severe moral injury disrupts pretty primary senses of the self and the senses of our inner personal self that develop … like a sense of agency and physical cohesion, an ability to transmit meaning, to have inner subjectivity with others, a possibility of having inner subjectivity of having affectivity. You see dissociation, and numbness, and a sense of lost in time, and cosmic loneliness.” (FG6, research and clinical care expert 30).

SMEs emphasized that clinically recognizable symptoms are not required for moral injury; some individuals suffer in ways that may be overlooked by standard assessments:

“I will say that many veterans who are suffering from moral injury don’t necessarily meet criteria for PTSD or depression, but they still struggle with suicidality, hopelessness, isolation. A lotta those veterans often fall through the cracks because their injury doesn’t look clinical.” (FG1, research, clinical, spiritual care and healthcare ethics expert, veteran 1).

Moral injury’s subjective nature, similarity with other psychosocial-spiritual constructs, and symptom expressions that can be difficult to quantify (e.g., hopelessness, helplessness, unworthiness) create challenges to identifying when someone is affected.

### 3.2 Domain II: implications for moral injury identification and care

SMEs spoke about the practical and clinical utility of the moral injury construct and shared how these may impact the application of the construct across disciplines as well as inform intervention approaches. Analysis yielded three main themes.

#### 3.2.1 Theme 1: practical considerations for the use of the moral injury construct

SMEs noted that the term moral injury is often self-descriptive, helping individuals articulate experiences that might otherwise be difficult to express. While broadening the concept can empower people to describe their experiences across contexts, some cautioned that becoming too inclusive risks concept creep and diluting the specificity and impact of the term.

Several SMEs emphasized the value of broad, multi-level definitions of moral injury, noting that such definitions foster a deeper understanding of this complex phenomenon and allow for the inclusion of acute, chronic, environmental, systemic, and generational dimensions, while also providing opportunities to challenge societal stereotypes. A key utility of moral injury, as highlighted in the following quote, is its ability to bridge individual experiences with broader existential meanings, resonating in both clinical contexts and with the broader public:

“I think part of the reason that moral injury has taken off the way it has and resonates with people so deeply, whether it’s clinicians or the public, is that it captures the more existential piece, the meaning piece, the responsibility piece, the what does this say about me; what does this say about my role in the world? I think that’s really special and unique about moral injury.” (FG2, research and clinical care expert 10).

SMEs expressed mixed opinions on existing measures of moral injury, with the high-level reflective functioning required noted as a limitation. However, another expert described the utility of these measures from a different perspective:

“We see that 30 to 50 percent of people will report that they witnessed or participated in something they thought was wrong. A much smaller percentage will screen positive for what we call a functionally impairing level of moral distress.” (FG2, research expert 9).

#### 3.2.2 Theme 2: diagnostic considerations for the use of the moral injury construct

Opinions varied regarding the perceived value of including moral injury as a formal psychiatric diagnosis. Concerns included challenges with its identification (e.g., overlap with other mental health issues and variations in presentation), the potential to exclude context-specific and cultural experiences, and the risk of placing disproportionate responsibility on the individual to recover from a diagnosis rather than addressing systemic responsibility. Regarding the latter, several SMEs cautioned that diagnostic labeling risks pathologizing a person’s moral response to ethically compromised environments, thus obscuring accountability at the organizational, societal, or other systems-level. Additionally, some SMEs raised concerns over the broader risk of pathologizing moral emotions at large, emphasizing that moral pain may reflect intact moral functioning rather than psychopathology:

“That’s why I’m loathed to ever consider moral injury a diagnosis. I think we run the risk of pathologizing moral pain, which is an entirely natural, nonadaptive human experience. I think that the healing process entails learning to hold moral pain differently. It’s not just a healing process. It’s a growth process. It’s a personal evolution process of learning to be with these really powerful socially important emotions and in an enriching way, not a disruptive way.” (FG4, research and clinical care expert 19).

One participant hypothesized that individuals with moral injury who are experiencing functional impairment will likely qualify for an existing diagnosis, and that moral injury themes should be considered rather than diagnosing with moral injury:

“I would imagine there’s—whether it’s major depressive disorders or PTSD, substance-use disorder. We think about these existing categories. For many people that have developed functional issues related to a morally injurious event, more times than not, they’re gonna fit into one of these clinical categories. Then I think we just need to be aware of moral-injury themes within the context of these existing categories that we already have.” (FG6, research and clinical care expert 33).

This perspective was not shared by all SMEs. Some experts noted that individuals with moral injury may get missed and not qualify for services if they do not meet criteria for another diagnosis.

When asked specifically about incorporating moral injury into the Diagnostic and Statistical Manual of Mental Disorders (DSM), some SMEs strongly opposed the idea, noting that the manual already includes too many disorders and that adding more could weaken its construct validity, thereby reducing its utility and value. A couple of SMEs expressed some functional utility to including moral injury in the DSM as a means to provide a diagnosis and bill for services. As one expert stated:

“I think that there should be a way for people to get care for moral injury if they don’t meet criterion A for PTSD. They’re traumatized, but they need to get care. If we can utilize some of these codes and revise what we have, we might be able to get people the care they need without shoehorning this into a diagnostic system.” (FG6, research and clinical care expert 30).

SME perspectives overall position moral injury as a construct with clinical relevance that may be best understood as transdiagnostic, raising questions about whether its utility lies in formal classification or in its capacity to inform assessment, prevention, and care.

#### 3.2.3 Theme 3: recommended care and intervention for moral injury

**Figure 2:**
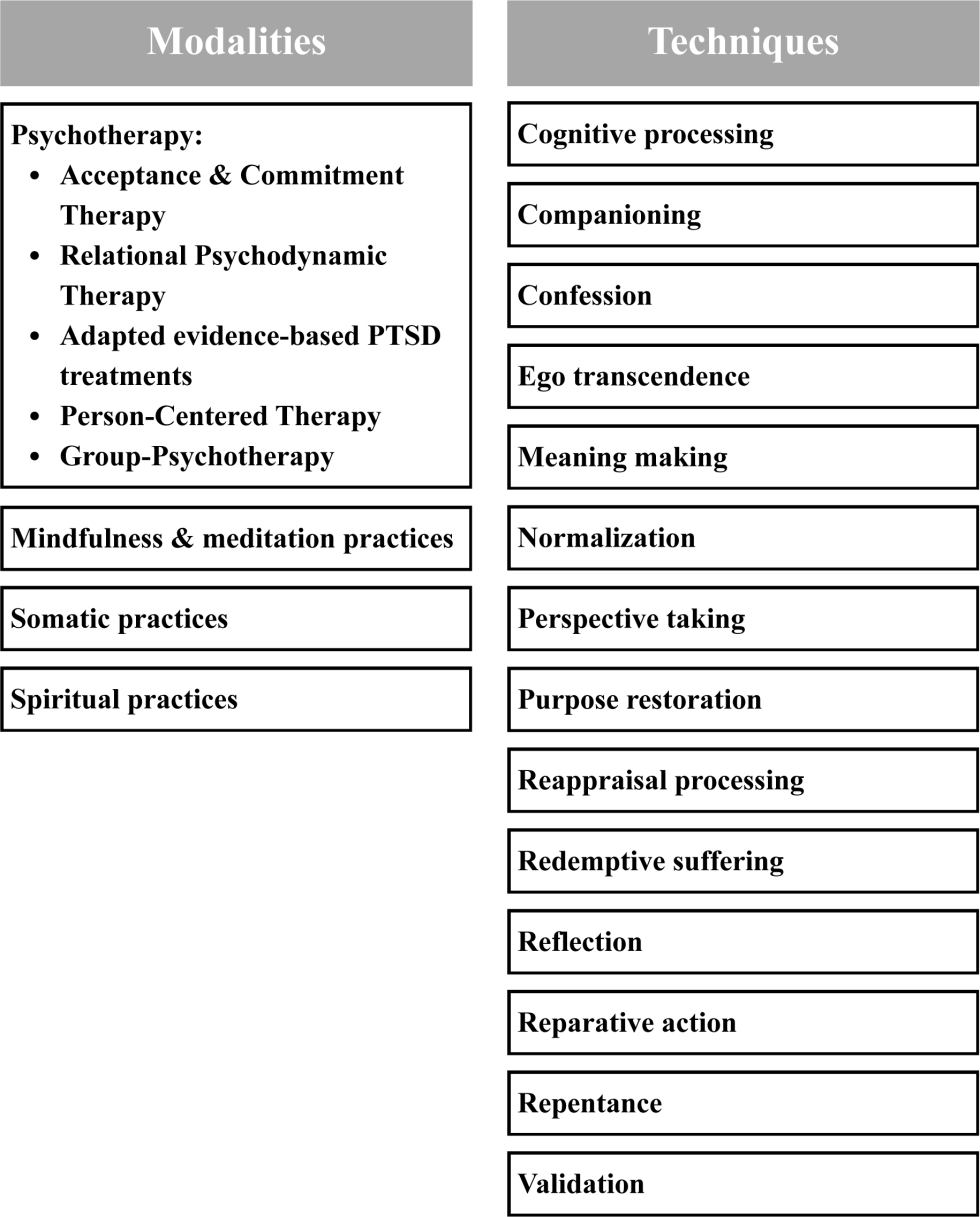
Recommendations for moral injury intervention and care provided by subject matter experts.

Given the complex, subjective, and multidimensional nature of moral injury, SMEs emphasized the importance of flexible, individualized care that integrates systemic, community, relational, individual, and spiritual approaches. Numerous specific treatments, intervention modalities, and techniques were noted (see **Figure 2**).

Importantly, it was highlighted that in order to effectively support healing and restoration, interventions must be tailored to: (1) type of moral injury (e.g., perpetration- versus betrayal-based); (2) individual manifestation of distress (e.g., the presence of specific emotions and/or behaviors, including internalization/externalization, social disruption, identity disruption, or dissociation); (3) psychological capacities (e.g., alexithymia, insight, self-awareness), and (4) individual meaning-making framework (i.e., individual’s belief system shaping their perception of the experience).

While there was no full consensus on the foundations of moral development or the mechanisms through which moral injury operates, there was broad agreement across SMEs that care should not be confined to a single type of provider. SMEs emphasized the importance of a multidisciplinary approach, bringing together clinicians, chaplains, researchers, and other professionals as essential to effectively addressing moral injury and mitigating its profound impact on individuals’ lives. One expert, also a veteran, explained:

“When I first started my work with moral injury, I didn’t really understand the extreme connection to upstream suicide prevention that I do today. This work is upstream preventing suicide. When I did a deep dive into diagnostic and nondiagnostic criteria for suicide, they were all drivers to isolation and loneliness, which are the two worst risk factors [of suicide] … I would argue, to this panel and probably ‘til the day I take my last breath, that we’re losing more veterans, more people, to morally injurious situations than we are anything diagnostic. It doesn’t get the attention because it’s not necessarily that scientific—I agree, 100 percent … that this is an all-hands-on-deck approach. We need every type of professional in the trenches with this because, if we really wanna put a dent in suicidality among veterans, … humans, we have got to pay attention to moral injury, 100 percent.” (FG1, research, clinical, spiritual care and healthcare ethics expert, veteran 1).

SMEs expressed the perceived benefit of having a variety of evidence-based interventions available for moral injury that could be integrated by different practitioner groups, including psychologists, social workers, chaplains, clergy, community-based groups, and peer groups. One expert shared:

“My hope in this space is because of the complexity of morality, and culture, and context, and religion that we really move into a space where there’s a dozen or more of these interventions that we consider good enough from an evidence perspective to say, “Here’s some community interventions over here. Here’s psychologists over here. Here’s a social worker. Here’s a chaplain. Here’s a clergy member. Here’s just a peer group.” (FG6, research, clinical and spiritual care expert 31).

The importance of disseminating services across settings, avoiding siloed care, and reaching individuals who may seek support outside of traditional medical clinics was emphasized. Several SMEs highlighted the need for cross-sector training; for example, one research and clinical care expert expressed the importance of helping psychologists “expand their frame of reference to attend to spiritual/religious aspects of people’s lives” (FG6, research and clinical care expert 33). Another expert noted that clinicians play a crucial role in baseline screening for moral injury and recommended broader adoption of multidisciplinary recovery teams. Together, these perspectives underscore the value of integrated, cross-disciplinary approaches to ensure comprehensive and accessible care for individuals affected by moral injury.

Across all focus groups, SMEs emphasized that addressing moral injury requires approaches that extend beyond traditional psychotherapy, integrating individual, relational, spiritual, and community levels of care. Multidisciplinary collaboration and flexible models were described as essential to effectively supporting individuals, communities, and even entire systems impacted by moral injury.

## 4 Discussion

This qualitative study provides empirical support for the context-dependent nature of moral injury while also illuminating shared foundational elements across disciplines. In examining how SMEs across fields and discourses conceptualize and work with moral injury within their domains of expertise, the present findings advance scientific understanding of moral injury, inform ongoing efforts to refine conceptual models, and offer guidance for assessment and intervention. Overall, the results suggest that no single model fully encompasses the complexity of moral injury as it is currently applied; however, substantial convergence exists around several core assumptions that may serve as the foundation for a more cohesive paradigmatic framework.

Across disciplines, SMEs primarily conceptualized moral injury through a phenomenological lens, emphasizing subjective interpretation and individual meaning-making within systems-based (i.e., relational, institutional, and sociocultural) contexts. Moral identity emerged as critical construct for establishing a foundation for conceptualizing moral injury. Despite its central role, SMEs noted that moral identity itself is fraught with conceptual challenges. These observations align with a recent systematic review of 26 morality studies, which concluded that no single model fully explains morality (27). The review referenced multiple salient conceptual approaches (i.e., innate moral sense, moral development shaped by social relationships, and environmental influences), and, like our study, revealed that, despite differing perspectives, models share a core principle that moral sense is innate but shaped and reinforced by social and environmental context. Similarly, SMEs in the present study drew on evolutionary, biological, relational, spiritual, and sociocultural frameworks to explain how moral identity develops and may be disrupted.

As illustrated in **Figure 1**, SMEs described the development of moral injury as beginning with exposure to a morally salient experience, which was interpreted through a moral/values/identity-based lens. These exposures involve a moral dilemma marked by active (perpetration-based) or passive (betrayal-based) violations of moral expectations that may result in a breach of trust and emotional distress. However, given subjectivity in how PMIEs are experienced and interpreted, and the lack of an objective threshold for determining at which point distress constitutes moral injury, most SMEs agreed that neither PMIEs nor moral distress alone are sufficient defining criterion for moral injury. Rather, SMEs described moral injury as a developmental, identity-level process. According to SMEs, moral injury is not defined by particular behaviors, emotions, or cognitive processes, but rather by the sustained disruption to a person’s moral identity, relational trust, and capacity for meaning-making that may manifest in impaired moral, relational, spiritual, psychosocial, or behavioral functioning. This conceptualization closely aligns with core assumptions articulated in social-functional models, particularly the emphasis on moral injury as a breakdown in moral and relational repair rather than a fear-based trauma response (28). SMEs’ descriptions strongly supported the view that moral injury exists on a continuum, varies in severity and functional impact, and does not require pathologization to warrant care. At the same time, SME perspectives extended beyond a purely social-functional framing, echoing key tenets of developmental and cognitive models (19, 20), where moral injury is shaped by prior experiences, moral schemas, and relational histories. Such frameworks emphasize moral distress as a normal and appropriate response to moral transgression and betrayal, with impairment emerging when preexisting schemas, maladaptive metacognitions, or contextual constraints interfere with integration of new moral contexts and reparative processes. SMEs across disciplines reinforced that moral emotions, such as guilt and shame, are not inherently pathological, yet can become injurious when overwhelming, suppressed, or disconnected from opportunities for accountability, repair, and redemption.

SMEs converged on the necessity of exposure to a morally salient experience as a precondition for moral injury, consistent across all existing major frameworks. However, there was meaningful divergence in how such exposures were defined and weighted. While early clinical and military-based models emphasize acute, high-stakes events involving life threat (2), some SMEs challenged the sufficiency of this framing. Many highlighted cumulative, chronic, and systemic moral harms that do not involve events modeled after criterion A for PTSD but nonetheless erode moral identity and trust over time. This broader conceptualization aligns closely with developmental (19, 20), biopsychosocialspiritual (21), and structural models of moral injury (22). SMEs emphasized that morally salient exposures occur within relational and systemic contexts, often shaped by institutional cultures, power hierarchies, and social inequities. Betrayal-based exposures, particularly those involving leadership failures, institutional neglect, or systemic injustice, were viewed as central yet historically underrecognized PMIEs. This resonates with structural models that locate moral harm within collective systems that emphasize shared responsibility, as well as with biopsychosocialspiritual frameworks that emphasize betrayal, meaning, and existential rupture as defining features of moral injury. This shift illustrates the evolution of the moral injury construct toward growing recognition that moral injury may not necessarily be understood through individual experiences alone but rather through their intersection with broader contextual dimensions. Supporting this perspective, a recent interdisciplinary review of 84 moral injury studies identified societal, political, and organizational dimensions as critical areas warranting deeper exploration to inform effective approaches to intervention (29).

The field has made meaningful efforts to operationalize moral injury by assessing PMIEs and associated outcomes through instruments like the MIES, MIOS, and MIDS. Although these scales represent an important step forward in assessing moral injury, their broad application prior to establishing a formal definition and validation of the moral injury construct has been cautioned. Critiques of existing measures include their limited assessment of passive exposures and potential confounders, such as recent life stressors, functional problems, and response biases (30). While these measure-specific concerns were not widely echoed by focus group SMEs, their discussions nonetheless reinforced broader challenges associated with the objective assessment of moral injury. In particular, SMEs highlighted difficulties in clinical identification, emphasizing that moral injury cannot be determined by symptom frequency or severity alone. Examples shared suggest that many relevant symptoms do not align with traditional clinical presentations, and are highly subjective in meaning, raising concerns that conventional clinical measures may under-identify moral injury and potentially exclude many affected individuals from care. Consistent with biopsychosocialspiritual conceptualizations of moral injury (21), SMEs therefore emphasized the importance of a transdiagnostic approach to moral injury assessment, in which psychological, cultural, and spiritual dimensions are considered by care providers (e.g., clinicians, chaplains, etc.). Since the completion of the present study, the DSM-5-TR (31) has formally included the Z-code *Moral, Religious, or Spiritual Problem* (Z65.8) (32), providing clinicians with a mechanism to document morally relevant concerns without assigning a psychiatric diagnosis. Although this Z-code was not incorporated at the time of data collection, thus not explicitly referenced by SMEs, its inclusion reflects growing recognition of moral concerns within clinical science frameworks.

The complexity of objectively assessing moral injury, as well as its transdiagnostic nature, necessitates the need for multi-level, tailored, and context-sensitive intervention approaches according to SMEs. Discussions revealed the importance of multidimensional systems of care that move beyond traditional clinical models by integrating community and spiritual intervention when appropriate. Similarly, there is no one-size-fits-all intervention modality, with SMEs recognizing that, for care to be effective, it needs to be tailored to meet the needs of each individual. Given the variability in how individuals may present with moral injury and the multitude of possible intervention approaches, SMEs emphasized the need for cross-sector training and collaborative care models integrating spiritual and healthcare practitioners. The VA Integrative Mental Health initiative was provided as an example of an existing, integrated approach focused on cross-sector collaboration. The national initiative aims to establish a collaborative system of care for veterans, service members, and their families (33). The initiative brings together clinicians, spiritual care providers, and community members through education and outreach activities and evidence-based training opportunities to promote dynamic person-centered care. By equipping care professionals across diverse disciplines with the skills to recognize the signs of moral injury and creating cross-sector networks, SMEs expressed that people experiencing moral injury may be more likely to receive care that is effective, timely, and suited to their individual needs.

In summary, insights from SMEs across disciplines enhance our understanding of the growing relevance of the moral injury construct across diverse fields and populations. Focus group discussions revealed common features of moral injury, highlighting foundational assumptions that hold across disciplines. Conversely, the diversity of perspectives also illuminated areas of disagreement, underscoring the important need for a systematic approach to operationalize moral injury to ensure it is rigorous yet flexible.

### 4.1 Strengths and limitations

There are several notable strengths to this study. First, the study was conducted as a collaborative effort between qualitative public health researchers and mental health experts experienced in moral injury research and intervention. This collaboration ensured that the study followed rigorous qualitative methods and reflected up-to-date scientific and clinical knowledge in moral injury. While identification of SMEs and the development of the focus group discussion guide was a collaborative effort, to limit any potential bias, data was collected and analyzed independently by the team’s qualitative experts. Additionally, the inclusion of SMEs with diverse professional backgrounds (e.g., research, clinical care, spiritual care) captured a breadth of perspectives. This is important given the rapid uptake of the construct across diverse populations and the need to determine if there are shared foundational principles supportive of a centralized conceptual framework. Lastly, the multi-phase analytic approach conducted by members of the qualitative research team allowed for reflexivity, rigor, and transparency in the development of themes.

Limitations of the study should also be acknowledged. First, the sample consisted of purposively sampled participants, many of whom were affiliated with the VA and embedded within military context. As this study aimed to inform VA practices, the perspectives represented may reflect assumptions common to these settings, which may not fully translate to civilian or non-Western populations. This potential limitation is underscored by the lack of clear consensus regarding the necessity of exposure to a high-stakes event preceding moral injury. Additionally, group dynamics, inherent in focus groups, may have influenced interactions and SME responses. Despite this, the study team determined that the focus group format was most appropriate, as it facilitates the sharing of diverse perspectives, stimulates in-depth conversation, and allows for more nuanced insights. To support this, focus group facilitators were trained by the team’s qualitative experts to promote equitable participation among SMEs. Notably, the distribution of participant expertise across focus groups yielded unanticipated benefits, with some SMEs noting that engaging with others’ perspectives generated new insights into their own conceptualization of moral injury.

### 4.2 Future directions

Findings from this study suggest several next steps for advancing moral injury research and care. First, future work should focus on refining and empirically testing clearer conceptual boundary conditions for moral injury. While SMEs demonstrated substantial convergence around core features—exposure to agentic and/or non-agentic acts that violate deeply held moral beliefs and expectations, often compounded by fractured trust, a failure of repair; identity and/or relational disruption; persistent moral distress or suppression of moral emotions; and significant functional impairment—there remains variability in how these elements are weighted, expressed, and experienced across contexts. Research should prioritize operationalizing these features from a bounded flexibility model, testing which features must be present for moral injury to be meaningfully distinguished from acute moral distress, including examining cases in which moral injury is present without consciously articulated moral pain. It is also important to note that the conceptual foundations of moral distress within nursing literature substantially inform current understandings of moral injury within healthcare contexts. While our study included SMEs with experience in healthcare settings, nurses were not represented in the sample. Given the depth of nursing scholarship on moral distress and its influence on contextual models of moral injury, future research should incorporate nurse perspectives to further refine conceptual boundaries and ensure alignment with established frameworks, as well as inform standards of care.

Second, research is needed to clarify developmental pathways from experiencing a moral violation to identifying with the construct of moral injury. Longitudinal and mixed-method designs may be especially valuable for examining how moral distress resolves adaptively versus when it evolves into moral injury, as well as how presentation and impact differ following acute, chronic, or systemic PMIE exposure. Efforts should also examine developmental distinctions between perpetration- and betrayal-based PMIEs, as well as subsequent care implications. Further, more focused investigation of moral repair processes is needed. SMEs highlighted that moral injury frequently persists not due to individual pathology, but because opportunities for repair are constrained; thus, future work should investigate the role of reparative processes within interpersonal, communal, organizational, and systemic contexts in mitigating moral injury. Understanding how repair is facilitated and obstructed across these levels is essential for informing effective prevention and intervention models. There is also a need for further research related to clinical training. Future studies should explore training models, supervision frameworks, and organizational practices that support clinicians to engage in moral injury care without over-pathologization, while also addressing structural barriers, promoting systemic responsibility, and employing dynamic, multidisciplinary approaches. Exploring the integration of moral injury-informed models into existing care systems and evaluating existing integrated care models is also warranted.

Finally, future research should center the perspectives of individuals with lived experience of moral injury. While expert consensus offers critical guidance, continued conceptual refinement and care innovation depend on incorporating the voices of those directly affected. Research that elevates lived experience is essential for ensuring that moral injury frameworks, prevention efforts, and treatment models are aligned with the realities of those they intend to serve.

## Data Availability

The datasets presented in this article are not readily available because data are owned by the U.S. Department of Veterans Affairs, which determines applicable data-sharing protocols. Requests to access the datasets should be directed to jason.nieuwsma@va.gov.
